# The Indiana Dental Journal

**Published:** 1898-04

**Authors:** 


### The Indiana Dental Journal.

This new enterprise entered upon its career January, 1898, and judging from the four numbers already at hand, and knowing the energy and the push of the editor, there can hardly be a doubt of its success.

Its pages are full of interesting and valuable matter. It should be in the hands of every progressive dentist. Each numbers contains about one hundred pages, and the general make-up is excellent throughout. May it success be equal to the expectations of its projectors.

